# Effects of Image Dataset Configuration on the Accuracy of Rice Disease Recognition Based on Convolution Neural Network

**DOI:** 10.3389/fpls.2022.910878

**Published:** 2022-07-05

**Authors:** Huiru Zhou, Jie Deng, Dingzhou Cai, Xuan Lv, Bo Ming Wu

**Affiliations:** College of Plant Protection, China Agricultural University, Beijing, China

**Keywords:** deep learning, convolutional neural network, rice diseases, image recognition, crop disease dataset, model fitting

## Abstract

In recent years, the convolution neural network has been the most widely used deep learning algorithm in the field of plant disease diagnosis and has performed well in classification. However, in practice, there are still some specific issues that have not been paid adequate attention to. For instance, the same pathogen may cause similar or different symptoms when infecting plant leaves, while the same pathogen may cause similar or disparate symptoms on different parts of the plant. Therefore, questions come up naturally: should the images showing different symptoms of the same disease be in one class or two separate classes in the image database? Also, how will the different classification methods affect the results of image recognition? In this study, taking rice leaf blast and neck blast caused by *Magnaporthe oryzae*, and rice sheath blight caused by *Rhizoctonia solani* as examples, three experiments were designed to explore how database configuration affects recognition accuracy in recognizing different symptoms of the same disease on the same plant part, similar symptoms of the same disease on different parts, and different symptoms on different parts. The results suggested that when the symptoms of the same disease were the same or similar, no matter whether they were on the same plant part or not, training combined classes of these images can get better performance than training them separately. When the difference between symptoms was obvious, the classification was relatively easy, and both separate training and combined training could achieve relatively high recognition accuracy. The results also, to a certain extent, indicated that the greater the number of images in the training data set, the higher the average classification accuracy.

## Introduction

Rice production is facing many threats, especially many diseases caused by fungi, bacteria, and environmental factors (Zhang et al., 2018). Timely and accurate diagnosis of rice diseases is critical to the management of these diseases. Traditionally, disease diagnosis was mainly done by experienced personnel based on visible symptoms and laboratory identification (Sethy et al., 2020). However, experienced personnel are in short supply at grass-roots plant protection stations in China and many other developing countries. Besides, the identification of crop diseases using laboratory technology is often laborious and time-consuming (Feng et al., 2020). Therefore, efforts have been made to develop alternative techniques, including image recognition based on machine learning for its timely feedback and low cost (Coulibaly et al., 2019; Abade et al., 2021; Bari et al., 2021).

Early automatic diagnoses of crop diseases were mainly done *via* image recognition based on traditional machine learning (Li et al., 2020). Many traditional machine learning algorithms, including self-organizing maps (Phadikar and Sil, 2008), back propagation neural network (Xiao et al., 2018), Naive Bayes (Islam et al., 2018), K-means clustering (Ghyar and Birajdar, 2017), and support vector machine (Yao et al., 2009), have been applied to the recognition of rice disease images. These algorithms achieved classification accuracy ranging from 92 to 97.2% in these studies, but the small training dataset and the huge feature extraction engineering have been two huge obstacles to the practical application of traditional machine learning algorithms in the field of rice diseases recognition (DeChant et al., 2017; Lu J. et al., 2017).

Deep learning, with the advantages of automatic feature extraction and efficient processing of big data, triggered a boom of research on image recognition these years (Min et al., 2017). Among many deep learning algorithms, the convolutional neural network (CNN) is most widely used in the field of computer vision (Voulodimos et al., 2018). The CNN automatically learns the features of the image through convolution and pooling operations, mimicking the processes of image recognition by the cerebral perception cortex (Yamins and DiCarlo, 2016), which suggested that CNN could perform like the human visual nerves in some way (Cadieu et al., 2014).

Recently, many researchers all over the world have also paid attention to apply deep learning, especially CNN, in the diagnosis of rice diseases. Some researchers trained existing CNN models with rice disease images (Ghosal and Sarkar, 2020; Deng et al., 2021; Krishnamoorthy et al., 2021), some built their own CNN models (Lu Y. et al., 2017), and some modified the classical CNN models such as DenseNet by adding inception module (Chen et al., 2020). Lightweight models, such as simple CNN in which model parameters were greatly reduced without precision loss, have also been developed for application with mobile devices (Rahman et al., 2020). As CNN is excellent in extracting features, Liang et al. (2019) also used a traditional SVM classifier for subsequent image classification based on image features extracted by CNN from images of rice leaf blast and achieved a significantly better classification accuracy by combining SVM with CNN than by combining SVM with two traditional feature extraction methods, namely, LBPH and Haar-WT.

The existing research results suggested that deep learning-based image recognition has become more and more mature and achieved high performance in the recognition of rice diseases, both in accuracy and efficiency. Therefore, instead of building new models or improving algorithms, more attention has been paid to solve specific and practical issues in training existing models by some researchers recently. For example, Mohanty et al. (2016) found that the image type used in model training and the image allocation ratio between the training set and test set would have effects on the diagnosis accuracy of the resulted model. Picon et al. (2019) proved that training a model for multi-crops performed slightly better than developing specific models for individual crops. Lee et al. (2020) proved that if a model was trained with datasets containing plant diseases that were not associated with a specific crop, the model would be more suitable for a wider range of uses, especially for images obtained in different fields and images from unseen crops.

Similarly, automatic diagnosis of rice diseases has encountered some practical problems because of the high complexity of rice disease symptoms under field conditions. For example, similar or different symptoms can develop at different stages, under different weather conditions, or on different plant parts. Previous studies on the diagnosis of rice diseases concentrated on the recognition of typical symptoms of different rice diseases, but rarely addressed how the images of different symptoms caused by the same disease should be tagged in the construction of the training dataset. Should they be divided into different classes or combined into a single class? How will the different data configurations affect the accuracy of models? This has become an urgent problem to be solved before the automatic disease diagnosis can really be applied to field conditions.

Therefore, taking rice blast and rice sheath blight as examples in this study, experiments were conducted to explore how the split or merged disease classes in the configuration of training databases affect the recognition accuracy of the model. The specific objectives of this study were as follows:

(1) To select an appropriate model from 5 common CNN models for the subsequent investigation;
(2) To evaluate the effects of three training data configuration methods on the performance of CNN models during the training and test processes;
(3) To identify where the misclassifications lie *via* constructing a normalized confusion matrix for each method; and
(4) To explore the possible causes for misclassification by visualizing the recognition process.

## Materials and Methods

### Choosing Crop Diseases and Construction of Datasets

#### Collection of Disease Images

Images of healthy rice leaves (HRL), and rice leaves or sheaths with symptoms of the three common diseases, rice blast (RB), rice brown spots (RBS), and rice sheath blight (RSB) were collected mainly from experimental rice fields in Panjin and Dandong cities of Liaoning Province, China. In addition, images were also collected from the greenhouse on the campus of China Agricultural University (CAU), from CAU experimental fields in Haidian District, Beijing, and from commercial fields in Wuyuan County of Jiangxi Province and in Lu’an City of Anhui Province. These images were photographed using smartphones or cameras following three rules: (1) avoid overexposure caused by direct sunlight; (2) ensure that the targeted lesion was in the center of the picture; and (3) avoid different disease symptoms in a single picture.

**FIGURE 1:**
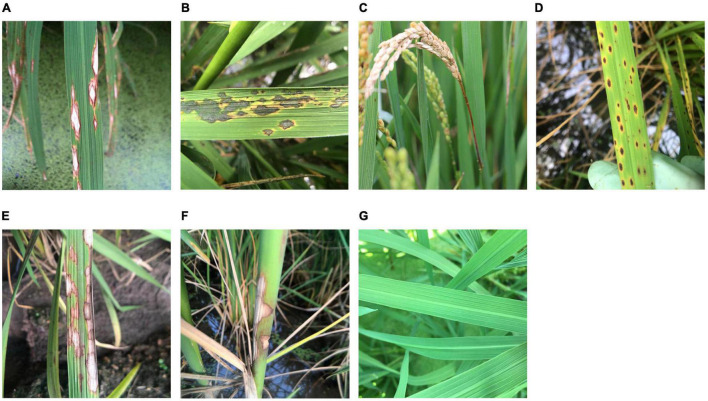
Sample images of healthy rice leaves and rice diseases. **(A)** Chronic lesions of rice leaf blast, **(B)** acute lesions of rice leaf blast, **(C)** rice neck blast, **(D)** rice brown spots, **(E)** rice sheath blight on leaves, **(F)** rice sheath blight on sheaths, and **(G)** healthy rice leaves.

The rice leaves, necks, heads, and whole plants with no visible symptoms were photographed and regarded as healthy rice plants. For rice leaf blast, images of chronic (RLBC), and acute (RLBA) leaf lesions were collected in this study at the early growth stage of rice because of their importance and prevalence under field conditions, while the other two less common symptom types, namely, white spot and brown spot, were not included in this study. According to Kato (2001), the chronic type leaf symptoms were defined as spindle-shaped leaf lesions with a yellow outside halo, a brown inner ring, and a gray white center (Figure 1A), while acute type symptoms were defined as the leaf lesions that are nearly round or oval in shape, which often become irregular, and look like water stains with a layer of dark green mold on the surface (Figure 1B). Besides, images of rice neck blast (RNB), the most economically important symptom of rice blast, were also collected at the late growth stages of rice in this study. According to Kumar et al. (1992), neck blast was defined as the symptoms that appeared around the neck of rice panicles as light brown spots at the initial stage and then gradually expand up and down, leading to a white gray color of the whole rice ear, and sometimes the death of whole ear (Figure 1C).

Since rice brown spot caused by *Bipolalaris oryzae* has a similar shape and yellow halo to those of rice blast leaf lesions, images of rice leaves with brown spots were collected and used to test the recognition accuracy of the outcome models. According to Quintana et al. (2017), the infected leaves with sesame-like oval dark brown spots surrounded by yellow halos were considered as typical symptoms of rice brown spot (Figure 1D).

Another important disease, rice sheath blight caused by *Rhizoctonia solani*, which can cause similar symptoms on leaves (RSBL) and sheaths (RSBS), was also included in this study to illustrate how the classification of similar symptoms on different plant parts caused by the same pathogen would affect the accuracy of recognition. According to Lee and Rush (1983), the typical symptoms of this disease are cloud-shaped lesions on the leaf sheaths and leaves, with brown to dark brown edges and grayish green to grayish white middle parts (Figures 1E,F).

#### Preprocessing of Images

As CNN requires squared input images, in order to avoid image deformation caused by the forced compression of non-squared images during input, the automatic clipping method was used to cut each image into a square, with the side length equal to the length of the short side of the original image and using the original image center as the clipping center. The clipped images were then compressed to 500 × 500 pixels. Subsequently, normalization was applied on each image by dividing all pixel values with 255 to accelerate the convergence of models during the subsequent training procedure.

As the number of acquired images in some classes was inadequate for model training and validation, more images in these classes were generated to meet the requirement by image augmentation (Table 1). The methods used in augmentation included flip, translocation, rotation, and zoom (Francois, 2018).

**TABLE 1:** The number of images within each disease class obtained in this study.

	Rice blast			Rice sheath blight				
	Leaf		Neck	Leaf	Sheath			
	Acute (RLBA)	Chronic (RLBC)	(RNB)	(RSBL)	(RSBS)	Rice brown spots (RBS)	Healthy rice leaves (HRL)	Total
Initial number	1,146	1,186	599	1,193	598	1,143	1,146	7,011
Number after augmentation	1,146	1,186	1,198	1,193	1,196	1,143	1,146	8,208

#### Experimental Scheme

Three experiments were designed to investigate the effects of dataset configuration on rice disease images recognition. In each experiment, two symptoms of one disease were selected for training and testing together with the other three diseases. In experiment 1, training datasets with separate and combined classes of RLBC and RLBA were compared. In experiment 2, training datasets with separate and combined classes of RLBC and RNB were compared. In experiment 3, training datasets with separate and combined classes of RSBL and RSBS were compared.

In each experiment, a method using two separated classes and two methods with one combined classes were compared. Considering that the imbalance of data may affect the training results, two methods were used in the construction of the combined class, directly combining all the images of two classes into one class, and randomly selecting half images from each class and combining them into one class.

#### Construction of Datasets

Images of each class were randomly numbered after preprocessing, with a unique ID for each image. For example, the first image of RSB was named “RSB (0).” For each class, the first 500 or 1,000 images were used in training and validation datasets as required, and the images 1,001–1,099 were used to build test sets.

There were three independent datasets for each experiment. In experiment 1, 1,000 images of each class were divided into training set and validation set according to a ratio of 8:2 for method A. In method B, 1,000 images of RLBC and RLBA were directly merged into one class, with twice as many images as the other classes. In method C, 500 images were randomly taken from RLBC and RLBA, respectively, to form a combined class. The same ratio of 8:2 was used dividing image data into constructing training and validation sets in both methods B and C. In addition to the 1,000 images, other 100 images of each class were randomly selected to form a 500-image test set. These 500 images were used to test all three methods A, B, and C, but classes of RLBC and RLBA would be merged into one class for testing methods B and C. In the same way, training, validation, and test datasets were constructed in experiment 2 and experiment 3 (Table 2).

**TABLE 2:** The number of images in each disease class in training experiments using different methods.

	Experiment 1			Experiment 2			Experiment 3		
Disease classes	Method A	Method B^b^	Method C	Method J	Method K	Method L	Method X	Method Y	Method Z
HRL^a^	1,000	1,000	1,000	1,000	1,000	1,000	1,000	1,000	1,000
RBS	1,000	1,000	1,000	1,000	1,000	1,000	1,000	1,000	1,000
RLBA	1,000	$\left[\begin{array}{c} 1,000\\ 1,000 \end{array}\right]$	$\left[\begin{array}{c} 500\\ 500 \end{array}\right]$	/	/	/	/	/	/
RLBC	1,000			1,000	$\left[\begin{array}{c} 1,000\\ 1,000 \end{array}\right]$	$\left[\begin{array}{c} 500\\ 500 \end{array}\right]$	1,000	1,000	1,000
RNB	/	/	/	1,000			/	/	/
RSBL	1,000	1,000	1,000	1,000	1,000	1,000	1,000	$\left[\begin{array}{c} 1,000\\ 1,000 \end{array}\right]$	$\left[\begin{array}{c} 500\\ 500 \end{array}\right]$
RSBS	/	/	/	/	/	/	1,000		

*^a^HRL, healthy rice leaves; RBS, rice brown spot; RLBA, rice leaf blast-acute lesions; RLBC, rice leaf blast-chronic lesions; RNB, rice neck blast; RSBL, rice sheath blight on leaves; RSBS, rice sheath blight on sheaths.*

*^b^The images from the two classes within the braces were combined into one single class for training.*

### Hardware and Software

Keras/Tensorflow backend framework based on Anaconda3 platform was used in this study (version: keras 2.2.4, tensorflow 1.15.0), and the training and validation processes were coded using Python 3.7 programming language. The computer was equipped with 32 g memory module and GTX 1080Ti graphics card. The computer operation system was the 64-bit Windows 10 professional edition. The programs were all run on a single graphic processing unit (GPU) because the training speed on GPU is much faster than that on the central processing unit (CPU).

### Training Parameter Setting

Instead of starting from scratch, transfer learning was applied in all model training experiments to saving training time by carrying the weights from the training on ImageNet dataset (Russakovsky et al., 2015). The learning rate was set as 0.001, and the training was run for 50 epochs with a momentum of 0.9, an optimization function of stochastic gradient descent (SGD), and a mini-batch size of 32.

### Model Selection

Different algorithms have their own purposes or specific application scenarios when designing or modifying. For example, a multi-stream residual network (MResLSTM) was designed for dynamic hand movement recognition (Yang et al., 2021), and a modified YOLO v3 algorithm was applied to detect helmet wearing by construction personnel (Huang et al., 2021). At present, however, there is no widely used model for the diagnosis of rice diseases, so we conducted a preliminary experiment to select from five representative CNN models for subsequent experiments on the construction of datasets.

The VGG series (Simonyan and Zisserman, 2014), first developed by the VGG group of Oxford University, were CNN models with stacked 3 × 3 convolution kernels for extracting complex features with a manageable number of parameters. Considering the moderate size of our disease data, VGG16 (16 layers) was selected as the representative of this model series. Compared with the VGG series, some CNN models used more network layers to extract higher dimension features and took different approaches to handle the gradient dispersion problem associated with deeper networks (Gao et al., 2019). Inception v3 was chosen as a candidate model in this study for its deep depths and its inception module, which uses convolution kernels of different sizes in the same layer to realize feature fusion of different scales and batch normalization to speed up the learning rate (Szegedy et al., 2015). ResNet50 (50 layers) was included as a representative of ResNet series, in which a residual module was introduced for a shortcut connection in the network allowing the original input information to be directly transmitted to the later layer (He et al., 2015). In addition, MobileNet v2 (Howard et al., 2017) and NASNetMobile (Zoph et al., 2018), two representatives of the current lightweight models in the application scenarios of mobile terminals or embedded devices, were also selected for their relatively excellent performance and small number of parameters (Wang et al., 2020).

A pre-experiment was conducted to compare the performances of the five CNN models in recognition of the three rice leaf diseases and healthy rice leaves (Figure 1G). The 1,000 training images from each of the five classes, namely, RLBC, RLBA, RBS, RSBL, and HRL, were divided into a training dataset and a validation dataset according to the ratio of 8:2. The models were trained for 50 epochs using the transfer learning method, and the initial weights of five models were all set as the shared weights from training on ImageNet as described in the “Training parameter setting” section. The size of models, speed of training (in seconds per epoch), the highest validation accuracy, the final validation accuracy, the average validation accuracy, and standard deviation of validation accuracy were used to evaluate the models.

### Experiments and Statistical Analysis

Subsequently, 3 experiments were done using the best model selected from the pre-experiment. Due to the random input order of mini-batches, the results of training could vary at each run. To estimate this variation and assess the reliability of the results, each of the three experiments was repeated three times. The final validation accuracy, final validation loss, test accuracy, and test loss were analyzed using the GLM procedure in SAS (version 9.4, SAS Institute Inc., Cary, NC, United States) to determine whether the effects of the training dataset configuration were statistically significant.

Over the 50 epochs of the training processes, the average validation accuracy and average validation loss of three repeated experiments were calculated every four epochs. As the performance of each method fluctuated over epochs in the training process, to better express the whole trend during the process, regression was performed to fit a negative exponential decay model to the average validation accuracy and an exponential decay model to average validation loss over the training processes for each method using the non-linear regression procedure in SAS (Version 9.4, SAS Institute Inc., Cary, NC, United States).

For validation accuracy, the following model was used:


$$A=A_{\max}-\left(A_{\max}-A_{0}\right)e^{\left(-r_{a}\cdot x\right)}$$

where *A* was the validation accuracy and *x* was the epoch number in training, while *A*_*max*_, *A*_0_, and *r*_*a*_ were parameters to be estimated in model fitting. *A*_*max*_ reflects the highest validation accuracy that the method can reach, *A*_*0*_ reflects the initial validation accuracy, and *r*_*a*_ can reflect the increase rate of *A* or improvement rate of validation accuracy over epochs.

For validation loss, the following model was used in regression:


$$L=L_{\min}+e^{\left(-r_{l}\cdot x+b\right)}$$

where *L* was the validation loss and *x* was the epoch number in training, while *L*_*min*_, *r_l_*, and *b* were parameters to be estimated during model fitting. *L*_*min*_ represents the lowest validation loss rate obtained by this method after unlimited epochs, *L*_*min*_ + *e*^(−*r*_*l*_ + *b*)^ reflects the initial validation loss at epoch #1, and *r*_*l*_ is related to the decline rate of validation loss.

After model fitting, the parameters were compared between different methods using Student’s *t*-test (Steel and Torrie, 1980) to characterize the disparity of the three methods in the training process.

### Normalized Confusion Matrix

Confusion matrix, which was widely used in the evaluation of classification accuracy in many areas, was constructed for comparison of different training dataset configuration methods based on test results. As the image numbers of the classes to be tested in this study varied among different training dataset configuration methods, to better reflect their difference in classification accuracy, the normalized confusion matrix was used. For any classification with *c* classes, the confusion matrix consisted of *c* rows × *c* columns, and the element in the *i*-th row and *j*-th column was calculated by dividing the number of images that belonged to the *i* class and were classified into the *j* class with the total number of images in the row.

### Heatmap

To understand which parts of the input image, such as the lesion edge, the center, or other areas, had contributed more to the automatic classification by the models, for each representative image with a high frequency of misclassification in recognition, a heatmap of class activation was generated using the GRAD-CAM algorithm (Selvaraju et al., 2020), in which the pixels that contributed heavily to the final classification will be presented as yellow to red colors and those that contributed less will be presented in green to purple colors. The heatmap generated this way serves as a tool for visualization of the feature extraction process of deep neural network.

## Results

### Performance of the Five Models in Pre-experiments

The results from the pre-experiment demonstrated that the five CNN models performed differently in the classification of these images (Table 3). VGG16 and Inception v3 all achieved a validation accuracy higher than 99%, but ResNet50 had the highest average validation accuracy and a smaller standard deviation among these models, suggesting that its convergence speed was the fastest and its performance was the most stable. Considering ResNet50’s excellent performance in training, including good speed (38 s/epoch), the highest final validation accuracy, the highest average validation accuracy, and the smallest standard deviation, it was selected for the subsequent training experiments.

**TABLE 3:** Performance of five CNN models in the classification of rice disease images.

CNN models	Seconds/epoch	Highest validation accuracy^a^	Final validation accuracy^b^	Average validation accuracy^c^	Standard deviation of validation accuracy^d^
VGG16	36	0.9900	0.9890	0.8758	0.2473
Inception v3	60	0.9980	0.9920	0.9803	0.4020
**ResNet50**	**38**	**0.9940**	**0.9920**	**0.9851**	**0.0107**
MobileNet v2	37	0.9830	0.9520	0.7138	0.2199
NASNetMobile	57	0.9870	0.9870	0.9693	0.0352

*^a^The highest validation accuracy achieved within the first 50 epochs.*

*^b^The final validation accuracy after 50 epochs.*

*^c^The average validation accuracy over the first 50 epochs.*

*^d^The standard deviation of validation accuracy over the first 50 epochs, which reflects the convergence rate of five models.*

*The training results of the selected model ResNet50 were shown in bold.*

### Experiment 1: Different Symptoms of the Same Disease on the Same Part

**FIGURE 2:**
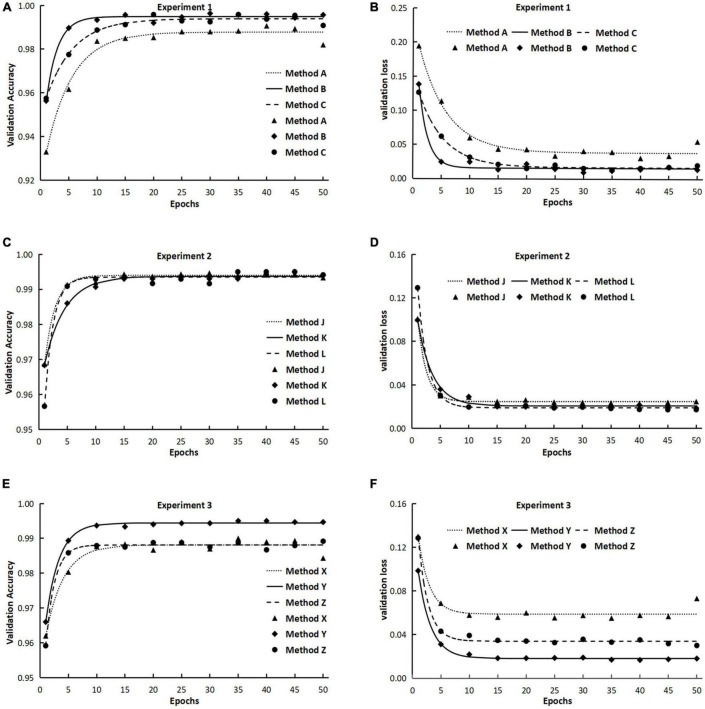
The validation accuracy and validation loss during the training processes in experiments 1, 2, and 3. (The points in the figures were means from three repeated runs, and the lines represented the fitted models of validation accuracy and validation loss.) Method A: Training with two separate classes, namely, acute type of rice leaf blast (RLBA) and chronic type of rice leaf blast (RLBC). Method B: Combining RLBA and RLBC as one class for training and the total number of images in the combined class was two times as those in the other three classes. Method C: Combining RLBA and RLBC as one class for training and the total number of images in the combined class was equal to those in the other three classes. Method J: Training with two separate classes of RLBC and rice neck blast (RNB). Method K: Combining RLBC and RNB as one class for training and the total number of images in the combined class was two times as those in the other three classes. Method L: Combining RLBC and RNB as one class for training and the total number of images in the combined class was equal to those in the other three classes. Method X: Training with two separate classes of rice sheath blight on leaves (RSBL) and rice sheath blight on sheath (RSBS). Method Y: Combining RSBL and RSBS as one class for training and the total number of images in the combined class was two times as those in the other three classes. Method Z: Combining RSBL and RSBS as one class for training and the total number of images in the combined class was equal to those in the other three classes.

The results from experiment 1 revealed that the training curves of validation accuracy and validation loss using method A differed from those using methods B and C (Figures 2A,B). Method A consistently had lower validation accuracy and higher validation loss than methods B and C did during the whole training process over 50 epochs (Figures 2A,B). Differences also existed between method B and method C in the early epochs of the training process, but the difference gradually decreased to an ignorable level with the increase of training epochs. Regardless of methods used in training dataset configuration, the trends of validation accuracy over training epochs could be fitted well to the negative exponential decay model (Table 4A) and those of validation loss fitted well to the exponential decay model (Table 4B). The *t*-test indicated that the highest accuracy (*A_max_*) obtained using method A was lower than those using the other two methods, while the lowest validation loss (*L_min_*) using method A was significantly greater than those using methods B and C (Tables 4A,B). The growth rate *r*_*a*_ of validation accuracy and the decline rate *r*_*l*_ of validation loss were significantly faster for method B than for the other two methods.

**TABLE 4A:** Parameters and determinant coefficients of models [*A* = *A*_*max*_−(*A*_*max*_−*A*_0_)*e*^−*r*_*a*_⋅*x*^] fitted to the validation accuracy over training epochs in 3 experiments using different methods.

Experiment-method	*A*_*0*_	*A*_max_	*r*_a_	*R* ^2^
1-method A	0.9191 ± 0.0047^b^	0.9878 ± 0.0011^b^	0.2154 ± 0.0289^b^	0.977
1-method B	0.9319 ± 0.0050^b^	0.9949 ± 0.0005^a^	0.4896 ± 0.0667^a^	0.985
1-method C	0.9489 ± 0.0028^a^	0.9939 ± 0.0007^a^	0.2077 ± 0.0254^b^	0.985
2-method J	0.9507 ± 0.0035^b^	0.9940 ± 0.0003^a^	0.5377 ± 0.0718^a^	0.991
2-method K	0.9603 ± 0.0014^a^	0.9937 ± 0.0003^a^	0.2800 ± 0.0230^b^	0.992
2-method L	0.9223 ± 0.0099^c^	0.9935 ± 0.0005^a^	0.6564 ± 0.1332^a^	0.992
3-method X	0.9520 ± 0.0037^a^	0.9881 ± 0.0006^b^	0.3206 ± 0.0629^b^	0.960
3-method Y	0.9510 ± 0.0015^a^	0.9944 ± 0.0002^a^	0.4244 ± 0.0260^b^	0.997
3-method Z	0.9340 ± 0.0053^b^	0.9881 ± 0.0003^b^	0.6274 ± 0.0921^a^	0.992

*R^2^: Degree of coincidence between test data and fitting function. The closer the value of R^2^ is to 1, the higher the degree of coincidence is.*

*Within each experiment, the fitted value followed by different letters in the same column differed significantly at confidence level p = 0.05.*

**TABLE 4B:** Parameters and determinant coefficients of models [*L* = *L_min_* + e^(−*r*_*l*_⋅*x*+*b*)^] fitted to the validation loss over training epochs in 3 experiments using different methods.

Experiment-method	*L_min_*	*r*_*l*_	*b*	*R* ^2^
1-method A	0.0382 ± 0.0030^a^	–0.2020 ± 0.0242^b^	–1.6449 ± 0.0591^a^	0.980
1-method B	0.0156 ± 0.0016^b^	–0.6331 ± 0.1274^a^	–1.4649 ± 0.1325^a^	0.985
1-method C	0.0165 ± 0.0009^b^	–0.2226 ± 0.0126^b^	–1.9856 ± 0.0281^b^	0.991
2-method J	0.0246 ± 0.0006^a^	–0.6433 ± 0.0788^a^	–1.9319 ± 0.0816^b^	0.996
2-method K	0.0206 ± 0.0005^b^	–0.3797 ± 0.0360^b^	–2.1610 ± 0.0499^c^	0.991
2-method L	0.0189 ± 0.0005^b^	–0.5632 ± 0.0353^a^	–1.6410 ± 0.0382^a^	0.991
3-method X	0.0587 ± 0.0019^a^	–0.5010 ± 0.1536^a^	–2.1412 ± 0.1754^ab^	0.948
3-method Y	0.0181 ± 0.0004^c^	–0.4453 ± 0.0216^a^	–2.0777 ± 0.0266^b^	0.998
3-method Z	0.0339 ± 0.0009^b^	–0.5686 ± 0.0682^a^	–1.7911 ± 0.0734^a^	0.994

*Within each experiment, the fitted value followed by different letters in the same column differed significantly at confidence level p = 0.05.*

The ANOVA and multiple mean comparison revealed that on both validation and test datasets, the validation accuracy and test accuracy obtained using method A were significantly lower than those obtained using methods B and C, and the validation loss and test loss obtained using method A were significantly greater than those obtained using methods B and C (Table 5).

**TABLE 5:** The accuracy and loss obtained with validation and test datasets in three experiments using different methods.

Experiment-method	Val_acc	Val_loss	Test_acc	Test_loss
1-Method A	0.9831^b^	0.0530^a^	0.9573^b^	0.1680^a^
1-Method B	0.9928^a^	0.0211^b^	0.9767^a^	0.0685^b^
1-Method C	0.9912^a^	0.0254^b^	0.9727^a^	0.0957^b^
2-Method J	0.9928^a^	0.0282^a^	0.9593^a^	0.1223^c^
2-Method K	0.9918^a^	0.0251^a^	0.9547^a^	0.1586^b^
2-Method L	0.9919^a^	0.0236^a^	0.9420^b^	0.1884^a^
3-Method X	0.9867^b^	0.0610^a^	0.9400^a^	0.2174^a^
3-Method Y	0.9927^a^	0.0225^c^	0.9593^a^	0.1060^a^
3-Method Z	0.9871^b^	0.0376^b^	0.9527^a^	0.1263^a^

*Each of the values in the table was the mean from 3 repeated runs. Within each experiment, the means followed by different letters in the same column differed significantly at confidence level p = 0.05.*

**FIGURE 3:**
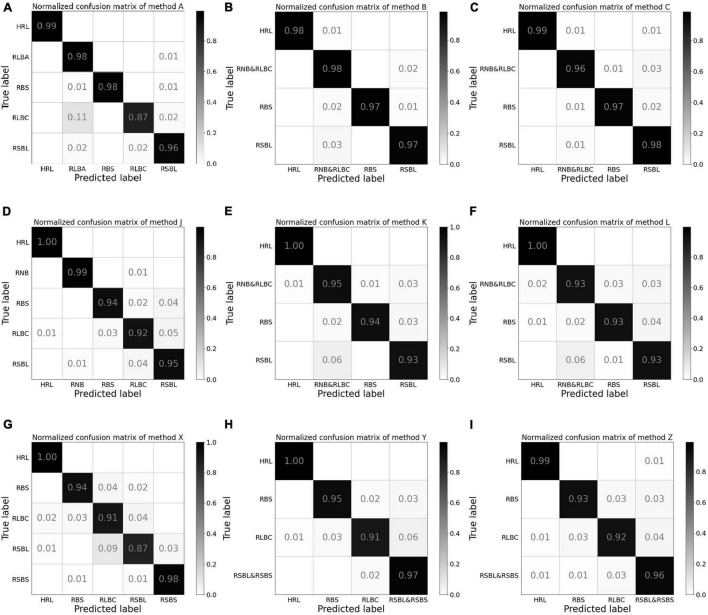
The normalized confusion matrix for the test results from experiments 1, 2, and 3. Method A **(A)**: Training with two separate classes, namely, acute type of rice leaf blast (RLBA) and chronic type of rice leaf blast (RLBC). Method B **(B)**: Combining RLBA and RLBC as one class for training and the total number of images in the combined class was two times as those in the other three classes. Method C **(C)**: Combining RLBA and RLBC as one class for training and the total number of images in the combined class was equal to those in the other three classes. Method J **(D)**: Training with two separate classes of RLBC and rice neck blast (RNB). Method K **(E)**: Combining RLBC and RNB as one class for training and the total number of images in the combined class was two times as those in the other three classes. Method L **(F)**: Combining RLBC and RNB as one class for training and the total number of images in the combined class was equal to those in the other three classes. Method X **(G)**: Training with two separate classes of rice sheath blight on leaves (RSBL) and rice sheath blight on sheath (RSBS). Method Y **(H)**: Combining RSBL and RSBS as one class for training and the total number of images in the combined class was two times as those in the other three classes. Method Z **(I)**: Combining RSBL and RSBS as one class for training and the total number of images in the combined class was equal to those in the other three classes.

The confusion matrix of test results using method A revealed that the class with the lowest accuracy was RLBC, and the main classification errors came from the misclassification of RLBC images into RLBA by the model (Figure 3A). When combining the two classes into one for training, the test accuracy ranged from 96 to 99% in every class with little variation among classes (Figures 3B,C). To understand why the misclassifications occurred, the original images of these misclassified RLBC images were visually examined again. It was found that although the leaf lesions in these images were nearly spindle shaped, the edges and corners were not obvious enough. When there were many lesions on leaves, they connected into pieces that were more like water stains, and the surfaces of some lesions were even gray green, which were typical symptoms of RLBA at the early stage of developing into RLBC (Kumar et al., 1992).

### Experiment 2: Different Symptoms of the Same Disease at Different Parts

The validation accuracy obtained using method K was highest among the three methods at the beginning of the training processes, and the lowest accuracy was gained using method L, but the accuracy increase rates *r*_*a*_ were higher for methods L (0.6564) and J (0.5377) than for method K (0.2800), and as a result, the three methods differed very less in accuracy after 20 training epochs (Figure 2C and Table 4A), and the maximum accuracy gained after 50 epochs varied from 0.9935 to 0.9940, showing no significant difference among the three methods (Table 4A). On the contrary, the validation loss using method L was the highest among the three methods early in the training, but it declined quickly as the training progressed and ended the training with a loss value that was very close to the other two methods (Figure 2D and Table 5).

Interestingly, although the accuracy using methods J, K, and L differed slightly (insignificantly) on validation data, the test accuracy obtained using method L was significantly lower, and the test loss was significantly greater than those obtained using method J and method K (Table 5).

The confusion matrix for method J in experiment 2 revealed that the model can distinguish RNB from other classes well, and the accuracy of RLBC was the lowest among the five classes, with majority of misclassification errors between RLBC and RSBL, but its recognition accuracy of RNB was relatively high (Figure 3D). When a combined class of RNB with RLBC was used in methods K and L, the accuracy of the combined RNB/RLBC class was between those of the two separate classes (Figures 3E,F). It was also noted that considerable errors existed in misclassifying RSBL into RLBC or combined class of RLBC with RNB regardless of the methods used (Figures 3D–F). This revealed that the identification of different plant parts is an indispensable part of classification by CNN models, and this identification could help to distinguish diseases on different plant parts, but similar symptoms on the same plant parts could not use this information and therefore become a more difficult task. It was also very interesting to note that method L had lower accuracy on combined RLBC/RNB class than method K. This might have been because method K had been trained with more images of the combined class than method L.

### Experiment 3: Similar Symptoms of the Same Disease at Different Parts

The initial validation accuracy of method Y was the highest among the three methods, and with the increase in training epochs, its validation accuracy remained highest all way to the end (Figure 2E). The results from *t*-test on model parameters *A*_*max*_ and *r*_*a*_ revealed that the highest validation accuracy *A*_*max*_ from method Y was significantly higher than those from the other two methods, but no significant difference in *r*_*a*_ was detected between method Y and method Z (Table 4A). The validation loss curves obtained with three methods displayed trends reverse to validation accuracy, in that no significant difference in the decline rate *r*_*l*_ of validation loss was detected among three methods (Figure 2F), but method Y had the lowest validation loss among the three methods, and method X had the highest validation loss.

The test results showed that the average test accuracy of method Y was much higher than that of method X and slightly higher than that of method Z (Table 5), although the ANOVA detected no significant difference between the three methods (see Supplementary Material). The confusion matrix of the test results illustrated that the model trained with method X misclassified 4% RLBC images as RSBL and 9% RSBL images as RLBC (Figure 3G). When the model was trained using method Y, with a combined class of RSBL&RSBS, its accuracy was greatly improved that it misclassified 6% of RLBC images into the combined class, but only misclassified 2% RSBL&RSBS images into RLBC (Figure 3H). Similar results were gained with method Z (Figure 3I). Once again, classifying between RLBC and RSBL was a difficult task, and the accuracy for the combined class was higher for method Y than for method Z, where more combined class images were used in training for method Y than method Z.

**FIGURE 4:**
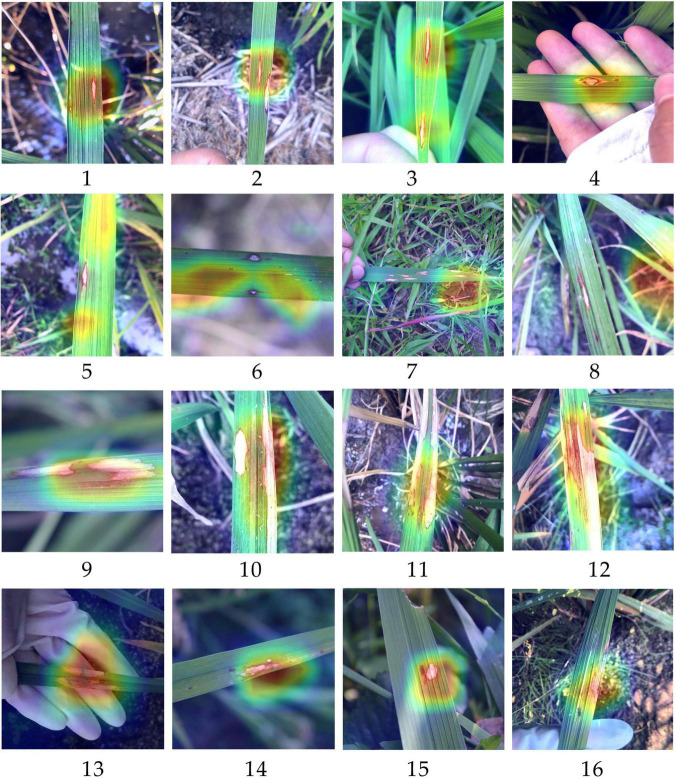
Heatmaps generated based on the classification by models trained with methods X for some rice leaf blast samples and rice sheath blight samples. **(1–4)** The samples of chronic type of rice leaf blast (RLBC) that were correctly recognized as RLBC; **(5–8)** The samples of RLBC that were mistakenly recognized as RSB. **(9–12)** The samples of rice sheath blight (RSB) that were correctly recognized as RSB. **(13–16)** The samples of RSB that were mistakenly recognized as RLBC. The red part has the highest contribution to the final prediction results. On the contrary, the purple part has the lowest contribution to the final prediction results of an image).

To further explore the reasons why differentiating RLBC and RSBL was difficult and easy to be misclassified for the outcome models, heatmaps of RLBC samples correctly classified, RLBC images misclassified as RSBL, RSBL samples correctly classified, and RSBL images misclassified as RLBC were compared (Figure 4). For those correctly classified RLBC, the areas with hot color were concentrated around the disease lesions, suggesting an excellent feature extraction by the model (Figures 4.1–4.4). However, it was observed that in most of the misclassified RLBC samples, the hot loci were not well overlapped with the disease lesions, suggesting the model didn’t extract important lesion features for decision-making, interfered either by other leaf damages (Figures 4.5,4.6) or field background (Figures 4.7,4.8). The existence of RLBC that directly led to the test results of the three methods of experiment 3 was not significantly different. Unlike RLBC, for all RSBL images, regardless of whether correctly classified or misclassified, the classification areas were mainly concentrated on the disease lesion area (Figures 4.9–4.16). It can be seen that compared with the typical symptoms of RSBL, in most of the misclassified samples, lesions were relatively small, had gray center areas, and were surrounded by brown halos, which was, to a certain degree, similar to the atypical RLBC, except for the subtle difference in lesion shapes (Figures 4.15,4.16). This may be one of the reasons why more rice sheath blight images were identified as RLBC than RBS.

## Discussion and Conclusion

In this study, we explored some specific problems encountered in dataset configuration for automatic recognition of rice diseases. The results from this study demonstrated that whether a combined class or several separated classes should be used depend on the similarity of these classes. For example, our results from experiment 1 demonstrated that using a combined class for RLBA and RLBC, two very similar symptoms on rice leaves, could achieve better recognition performance (higher accuracy and lower loss) than using two separate classes. A possible explanation might be that similar lesions sometimes are difficult to differentiate even for human experts because acute lesions often gradually develop into chronic lesions in the later stage (Kumar et al., 1992). This was also supported by the high misclassification rate between these two classes by method A using separate classes, but relatively low misclassification rates between any of these two classes and other class by method A. Similarly, for RSBL and RSBS in experiment 3, using a combined class in the training dataset could achieve a better performance than using two separate classes. A possible explanation is that using two separate classes of RSBL and RSBS will require the model to differentiate the similar cloud-shaped lesions on leaves and on sheaths and therefore will increase the possibility for the model to make mistakes in recognition of the background plant parts. However, using a combined class and using separate classes for RLBC and RNB had no significant impact on the performance of resulted models in experiment 2 where two symptoms were on different plant parts. A possible explanation for this was that with information from areas surrounding lesions, it is relatively easy for CNN model to differentiate two different symptoms, and thus, using a combined class or two separated classes didn’t have any significant impact on the final recognition as illustrated among methods J, K, and L in experiment 2 of this study.

The results from this study illustrated that a large number of images were required for training to achieve a high and repeatable recognition accuracy. As revealed in experiment 2, method L, in which the model was trained with half as many images of RLBC/RNB class as in methods J and K, although gained very high validation accuracy, performed significantly worse than methods J and K when tested with unseen images. So, for deep learning model, how many images are required to achieve best recognition effect? Is the more the number of images, the better the result will be? So far, few experts have explored this issue, and the number of images used in the existing literature varied from dozens to thousands. Rangarajan et al. (2018) discussed the influence of different number of images on the accuracy of the model, but the total number of images was small, and a scientific validation process has not been established yet. More in-depth studies are needed to answer this question in the future.

Through this study, we further prove the excellent ability of CNN in feature extraction. Based on the results of three experiments, it can be seen that the main features affecting the decision-making of rice disease classification models came from the disease lesion area, then from the area of plant organs, and finally from the image background. This is also consistent with the logic of human beings when classifying crop diseases. It can be seen from the heatmaps that for most samples, whether by correctly classified or by misclassified, the main feature areas that affect the model decision-making were still concentrated on the lesion area, and the areas were covered with red or yellow. The nearby areas of rice organs were also yellow, while the less important background areas were covered with blue or purple. The results of experiment 3 showed that when the disease lesions of RSB were similar, even if they existed on different organs, there would be confusion between RSBL and RSBS to some extent, indicating that the main distinguishing features still came from the lesion. At the same time, the results of experiment 2 showed that when the symptoms and organs were different, the model could extract more favorable information except the features of disease lesions, and that is why it can well distinguish the two classes when separately training RLBC and RNB. Does this mean that the image background is not important? Studies have shown that although the targets on the simple indoor background image and the complex field image were the same, the models trained by the two image sets could not be universal (Ferentinos, 2018). From the heatmaps of RLBC images misclassified as RSBL, it can be seen that although the error rate was low, the main factor causing the wrong model decision was the feature extraction of the field background. This also showed that the recognition of the background played an auxiliary role for the model. Therefore, it is very important to collect disease images under different conditions to improve the generalization ability of the models.

The results of three experiments showed that if the data configuration scheme was correct, the overall accuracy could be effectively improved. In experiment 1, combining the two similar leaf symptoms of rice and training, the validation accuracy was improved from 0.9831 to 0.9928, and the test accuracy was improved from 0.9573 to 0.9767, which was statistically significant at the confidence level of 0.05. Similarly, in experiment 3, the average accuracy of two symptoms of rice sheath blight was improved to 0.9700 from 0.9250 by using a combined class for similar symptoms on different plant parts. However, the results of experiment 2 revealed that for disparate symptoms on different plant parts, training with one combined class or two separate classes makes no difference, and the amount of data is a key factor affecting the overall accuracy. The average test accuracy of method L with a smaller data set was significantly lower (at a confidence level of 0.05) than that of the other two methods with larger datasets.

This study proposed a database configuration scheme among different symptoms of the same rice disease. Similar problems are often encountered in the diagnosis of other crop diseases (Barbedo, 2016). If our goal is to achieve a high overall classification accuracy, the findings from this study provide a reference. However, if the purpose is to differentiate multiple similar disease symptoms on different plant parts or at different stages, even if the symptoms are similar, they should be separately trained. Hopefully, the findings from this study can inspire researchers to put more efforts in automatic crop disease identification and think about the problems of disease identification from more different perspectives.

## Data Availability Statement

The original contributions presented in this study are included in the article/Supplementary Material, further inquiries can be directed to the corresponding author.

## Author Contributions

HZ led the whole experimental process and wrote the manuscript. JD provided suggestions about experimental design and guided the operation of the algorithms. DC participated in the construction of the dataset and collated data. XL participated in the image collection process. BW supervised the project and wrote the manuscript. All authors contributed to the article and approved the submitted version.

## Conflict of Interest

The authors declare that the research was conducted in the absence of any commercial or financial relationships that could be construed as a potential conflict of interest.

## Publisher’s Note

All claims expressed in this article are solely those of the authors and do not necessarily represent those of their affiliated organizations, or those of the publisher, the editors and the reviewers. Any product that may be evaluated in this article, or claim that may be made by its manufacturer, is not guaranteed or endorsed by the publisher.
