# Supplementary material for: Effects of Image Dataset Configuration on the Accuracy of Rice Disease Recognition Based on Convolution Neural Network
Source: Front Plant Sci. 2022 Jul 5;13:910878. doi: 10.3389/fpls.2022.910878 (PMC9295741; doi:10.3389/fpls.2022.910878)
Supplement: Supplementary file 1 [file Table_1.DOCX]

TABLE S1 | The validation results and test results of three experiments.

|  | val_acc | val_loss | test_acc | test_loss |
| --- | --- | --- | --- | --- |
| method A1 | 0.9826 | 0.0544 | 0.9500 | 0.2025 |
| method A2 | 0.9829 | 0.0564 | 0.9660 | 0.1233 |
| method A3 | 0.9839 | 0.0483 | 0.9560 | 0.1782 |
| Average method A | 0.9831 | 0.0530 | 0.9573 | 0.1680 |
| method B1 | 0.9921 | 0.0227 | 0.9760 | 0.0614 |
| method B2 | 0.9919 | 0.0242 | 0.9760 | 0.0856 |
| method B3 | 0.9944 | 0.0163 | 0.9780 | 0.0584 |
| Average method B | 0.9928 | 0.0211 | 0.9767 | 0.0685 |
| method C1 | 0.9914 | 0.0251 | 0.9860 | 0.0588 |
| method C2 | 0.9908 | 0.0269 | 0.9640 | 0.1413 |
| method C3 | 0.9914 | 0.0243 | 0.9680 | 0.0871 |
| Average method C | 0.9912 | 0.0254 | 0.9727 | 0.0957 |
| method J1 | 0.9947 | 0.0230 | 0.9660 | 0.1058 |
| method J2 | 0.9921 | 0.0292 | 0.9600 | 0.1283 |
| method J3 | 0.9916 | 0.0325 | 0.9520 | 0.1328 |
| Average method J | 0.9928 | 0.0282 | 0.9593 | 0.1223 |
| method K1 | 0.9906 | 0.0272 | 0.9520 | 0.1683 |
| method K2 | 0.9930 | 0.0254 | 0.9580 | 0.1411 |
| method K3 | 0.9918 | 0.0226 | 0.9540 | 0.1664 |
| Average method K | 0.9918 | 0.0251 | 0.9547 | 0.1586 |
| method L1 | 0.9913 | 0.0207 | 0.9380 | 0.1976 |
| method L2 | 0.9921 | 0.0233 | 0.9480 | 0.1834 |
| method L3 | 0.9922 | 0.0269 | 0.9400 | 0.1842 |
| Average method L | 0.9919 | 0.0236 | 0.9420 | 0.1884 |
| method X1 | 0.9855 | 0.0633 | 0.9240 | 0.3128 |
| method X2 | 0.987 | 0.0639 | 0.9440 | 0.1633 |
| method X3 | 0.9875 | 0.0559 | 0.9520 | 0.1761 |
| Average method X | 0.9867 | 0.0610 | 0.9400 | 0.2174 |
| method Y1 | 0.9932 | 0.0222 | 0.9640 | 0.1136 |
| method Y2 | 0.9927 | 0.0227 | 0.9680 | 0.0835 |
| method Y3 | 0.9921 | 0.0225 | 0.9460 | 0.1208 |
| Average method Y | 0.9927 | 0.0225 | 0.9593 | 0.1060 |
| method Z1 | 0.9867 | 0.0367 | 0.9600 | 0.1281 |
| method Z2 | 0.9871 | 0.0354 | 0.9500 | 0.1166 |
| method Z3 | 0.9874 | 0.0407 | 0.9520 | 0.1402 |
| Average method Z | 0.9871 | 0.0376 | 0.9540 | 0.1283 |
